# Clinical Characteristics and Manifestations of Fungal Esophagitis: A Single-Center Experience in South China

**DOI:** 10.1155/2021/8869494

**Published:** 2021-01-18

**Authors:** Shu-Kai Zhan, Xiao-Qin Wu, Feng-Ping Zheng

**Affiliations:** Department of Gastroenterology, The Third Affiliated Hospital of Sun Yat-Sen University, Guangzhou, China

## Abstract

Fungal esophagitis is a common infectious disease, although the pathogenic clinical characteristics remain incompletely clear, especially in South China. The goal of this study was to investigate the pathogenic clinical characteristics of fungal esophagitis and the efficacy of different therapeutic strategies at a tertiary hospital in South China. A retrospective study was conducted from January 2007 to December 2017. Data from 113,390 patients who were treated in the endoscopic unit were retrieved and analyzed. To further understand the pathogen and risk factors for fungal esophagitis, we performed a case-control analysis of 101 patients and 202 controls. Of the 113,390 patients, 932 (0.82%) were positive. The annual detection rate ranged from 0.345% to 1.106%, showing an initially increasing and subsequently decreasing trend. The patients' median age was 49 years (range from 8 to 85), and most were men (615/932, 65.99%). *Candida albicans* was found in samples collected from 36 patients, without any drug-resistant strains. Age (*P* = 0.018), malignancy (OR = 4.031, 95% CI: 1.562~10.407), cigarette smoking (OR = 3.017, 95% CI: 1.645~5.533), and the use of antibiotics (OR = 2.178, 95% CI: 1.078~4.400) or immunosuppressants (OR = 6.525, 95% CI: 1.089~39.105) were independently associated with esophageal candidiasis. Fluconazole had a better curative effect than nystatin (OR = 4.047, 95% CI: 1.282~12.772) or simple observation (OR = 8.91, 95% CI: 2.61~30.49). In conclusion, fungal esophagitis primarily affects men and elderly individuals; it develops in the setting of malignancy, smoking, and certain previous medication use. *Candida albicans* is the most common pathogen and is sensitive to antifungal agents. Fluconazole has a good therapeutic effect.

## 1. Introduction

Fungal esophagitis (FE) is an infectious disease caused by fungi adhering to and invading the epithelial cells of the esophagus [1]. As a result, white plaques, which are observable during gastroscopy, appear on the esophageal surface. Reports have shown that severe FE may cause esophageal hemorrhage, stricture or fistula formation, or even fungal-associated septicemia, accompanied by reduced quality of life and high mortality (approximately 20%-49%) in severe situations [2, 3]. A variety of symptoms including heartburn, acid regurgitation, nausea, dysphagia, and odynophagia have been reported to predict esophagitis, but which symptoms can predict FE remains unclear. The clinical presentations are nonspecific, and it is sometimes difficult to clinically or radiologically distinguish FE from other gastroenterological, cardiac, or neurologic diseases [4–7]. The risk factors for the development of FE in immunocompetent patients have not been entirely elucidated. In today's rapidly aging society, with the westernization of lifestyles and the widespread use of proton pump inhibitors (PPIs) and immunosuppressive agents, the pathogenic and epidemiological characteristics might vary. Studies on long-term trends of FE prevalence in South China have been very limited to date [8–11].

Therefore, in the present study, we report the results of a large, endoscopy-based, retrospective study and a case-control study aimed at investigating the epidemiology, clinical features, therapeutic interventions, and outcomes associated with FE.

## 2. Materials and Methods

### 2.1. Patient Selection

The study was approved by the ethics committee of the Third Affiliated Hospital of Sun Yat-sen University and was in accordance with the later amendments of the 1964 Helsinki Declaration. Before the case-control study began, we explained the purpose of the analysis to the FE patients in person or over the telephone and asked for their permission to use their physical data; all patients signed a consent form. Patients who refused were excluded from the study. Informed consent was exempted by the ethics committee of the Third Affiliated Hospital of Sun Yat-sen University for the retrospective study.

The general information and endoscopic diagnosis of 113,390 patients treated in the endoscopy unit between January 2007 and December 2017 in the Third Affiliated Hospital of Sun Yat-sen University were reviewed. Among them, 932 patients were diagnosed with FE. General information such as age, sex, and therapy strategy, as well as therapeutic efficacy, was collected and confirmed by medical records.

To further understand the pathogen and risk factors for FE, a case-control analysis was conducted from August 2018 to February 2019. Whenever FE was diagnosed, two other participants whose numbers were next to the FE patient were randomly checked simultaneously; these participants were defined as controls. General information such as the age, sex, and clinical data of the patients and controls was collected through questionnaires. Finally, 101 FE patients and 202 controls were enrolled after 9 patients and 18 controls were excluded for refusing or failing to complete the questionnaires. Each subject provided informed consent for participation in the study, and the research protocol was approved by the ethics committee of the Third Affiliated Hospital of Sun Yat-sen University.

### 2.2. Diagnosis of FE

The diagnosis of FE was made if white Candida plaques in the esophagus, as detected by endoscopy, could not be washed away, and fungal constituents, such as pseudohyphae or spores, were identified [5, 12]. The severity was evaluated by Kodsi's classification [13]. Decreased severity after treatment was regarded as effective. Kodsi's grading was determined by two experienced endoscopic doctors who were blinded to all patient clinical information. Whenever they had different opinions, a conclusion was made by another senior doctor.

### 2.3. Questionnaires

We obtained clinical data through a questionnaire that included questions about symptoms; lifestyle habits, such as cigarette smoking; history of medication use (PPI use, corticosteroid or other immunosuppressant use, and antibiotic use); and history of chronic diseases, specifically HIV, diabetes, cirrhosis, and malignant tumors. Overall, 101 FE patients and 202 controls completed the questionnaires in person or over the telephone.

The use of any PPI at a standard dose for at least 3 days was considered positive PPI use [14]. Similarly, the use of antibiotics for 3 days or a glucocorticoid at a dose equal to prednisone 20 mg was considered positive antibiotics or glucocorticoid use, respectively [11]. A patient with any history of smoking was regarded as a smoker [15].

### 2.4. Fungal Culture and Drug Sensitivity

Samples of white esophageal plaques were collected from 55 FE patients; the samples underwent fungal culture and type identification. The antifungal sensitivity of yeast pathogens was determined. At least two samples from every FE patient were taken with a disposable cytology brush (Alton, China) for analysis. The specimens were transferred to two Sabouraud glucose agar plates (Caring, China) and incubated at 28°C and 35°C simultaneously. Then, we identified Candida, filamentous or diphasic fungi after 24, 48, and 72 hours, respectively, according to the color and shape of the colony on the agar. When Candida was identified, the colony was transplanted to CHROMagar (Caring, China) to further confirm the subtype, as different Candida strains display different colors. The Candida colony was put into 0.85% saline, and an antimicrobial sensitivity test was performed with the ATB FUNGUS 3 method (bioMerieux S. A., France).

### 2.5. Statistical Analysis

The quantitative variable is presented as the median. For the comparison of qualitative data, such as the detection rate and clinical manifestations of FE, chi-square tests were performed. To analyze the associations, the Spearman rank correlation coefficient was calculated for ordinal variables, including the age group in the exploration of risk factors and the age stratification, initial disease severity, and recheck time period in the evaluation of therapeutic efficacy. For the multivariable analysis, a multiple logistic regression model was used to identify the different factors contributing to FE and therapeutic efficacy. The odds ratio (OR) and 95% CI were estimated. Two-tailed *P* values <0.05 were considered statistically significant. All analyses were conducted with the SPSS 20.0 software package (SPSS, Inc., Chicago, IL, USA).

## 3. Results

### 3.1. Detection Rate

**Figure 1:**
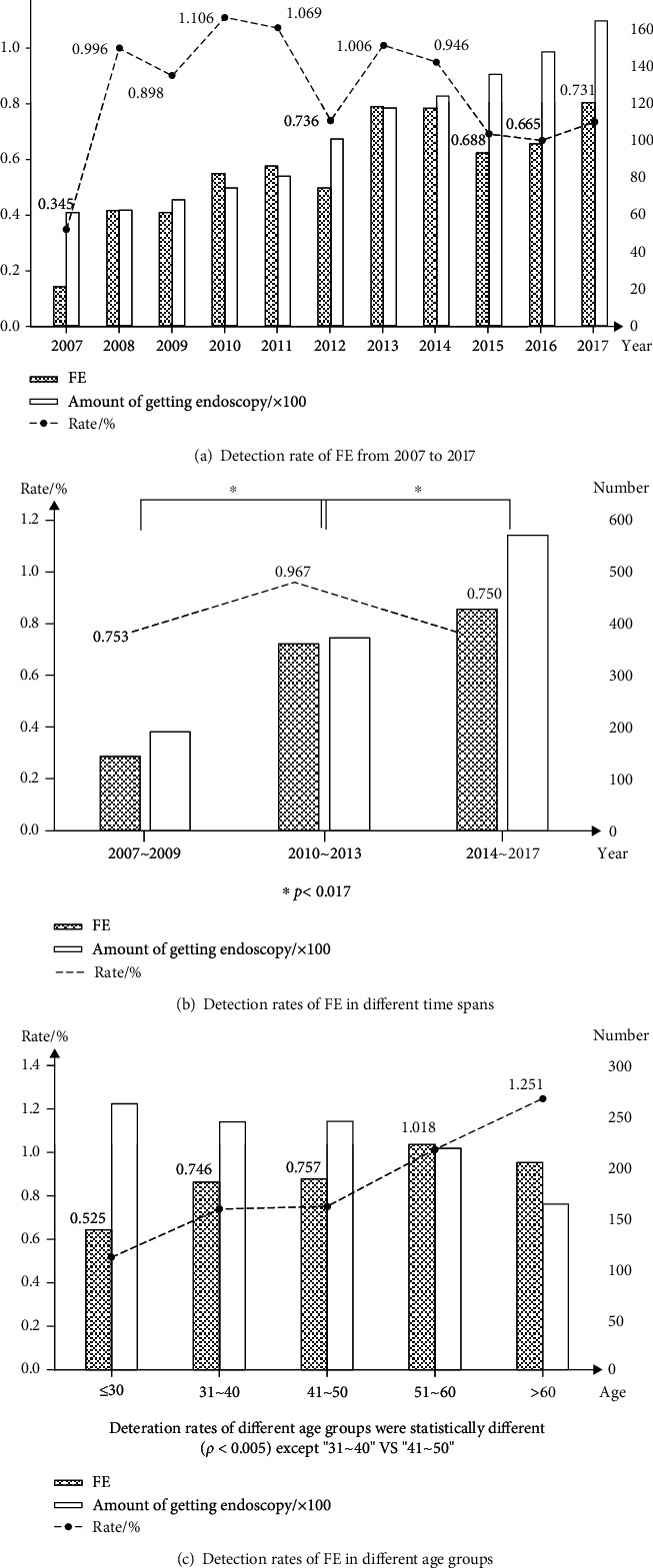
Detection rates of FE in different time spans and age groups.

From January 2007 to December 2017, 113,390 patients underwent gastroscopic examinations, among which 932 were diagnosed with FE. The overall detection rate of FE was 0.82%. The detection rate in each year ranged from 0.35% to 1.11% (Figure 1(a)). The highest rates were between 2010 and 2013; however, the rates decreased in the last 4 years (2007~2009 *vs.* 2010~2013: *χ*^2^ = 6.478, *P* = 0.011; 2010~2013 *vs.* 2014~2017: *χ*^2^ = 12.727, *P* < 0.001) (Figure 1(b)). Regarding sex, males seemed to be more susceptible to FE (615/932, 65.99%). The detection rate in males, which reached 1.0%, was higher than that in females, which was only 0.6% (*χ*^2^ = 48.97, *P* < 0.001). The median age of FE patients was 49 years old (range from 8 to 85). Among the different age groups, the detection rates varied, ranging from 0.55% to 1.25% (*χ*^2^ = 78.229, *P* < 0.001). Similar to other infectious diseases, elderly individuals were more susceptible to FE than young individuals. The detection rate in people over 60 years old was higher than those in the age groups of 31~40 and 41~50 years old, while it was lowest in the group under 30 years old (Figure 1(c)).

## 4. Clinical Manifestations and Risk Factors

In the case-control study including 101 FE patients and 202 controls, most of the FE patients were male (up to 70%) (FE *vs.* controls: *χ*^2^ = 6.48, *P* = 0.012), especially older males (FE *vs.* controls: Mann–Whitney *U* = 7547.0, *P* < 0.001). Regarding comorbidities, malignant tumor was more frequent in the FE patients than in the controls. Other comorbidities, such as HBV, cirrhosis, high blood pressure, and diabetes, were observed equally in both groups. Endoscopic comorbidities such as reflux esophagitis, bile reflux, esophageal varices, and esophageal polyps or papillomas were common. Among them, only esophageal polyps or papillomas were significantly different between the FE patients and controls; they were usually more common in the FE group.

Among all of the GI symptoms observed in the FE patients and controls, dysphagia and foreign body sensation were the most common in FE patients, while epigastric pain was the least common. Likewise, the ratio of smokers in the FE patient group was clearly higher than that in the control group. Moreover, the use of antibiotics or immunosuppressants seemed to contribute to FE. There was no significant difference in PPI use between the FE patients and controls (Tables 1 and 2).

A regression analysis was performed to analyze age; sex; smoking status; symptoms of dysphagia; globus sensations; epigastric pain; antibiotic use; immunosuppressant use; comorbidities of diabetes, malignant tumor, and esophageal polyps or papillomas; and FE incidence. As a result, increased age (*P* = 0.018), malignancy (OR = 4.031, 95% CI: 1.562~10.407), cigarette smoking (OR = 3.017, 95% CI: 1.645~5.533), and the use of antibiotics (OR =2.178, 95% CI: 1.078~4.400) or immunosuppressants (OR = 6.525, 95% CI:1.089~39.105) independently contributed to esophageal candidiasis. Esophageal polyps or papillomas (OR = 8.732, 95% CI: 2.006~38.013) and a foreign sensation when swallowing were also associated with the disease (OR = 1.507, 95% CI: 3.156~17.860) (Table 3).

## 5. Pathogen Identification

Samples of white esophageal plaques were collected from 55 FE patients from August to December 2018; the samples were subjected to fungal culture. *Candida albicans* was the causative agent for all 36 microbiologically confirmed cases. Specimen susceptibility testing against antifungal agents was performed. All of the specimens were sensitive to 5-flurocytosine, amphotericin B, fluconazole, itraconazole, and voriconazole.

## 6. Therapeutic Efficacy

In total, 157 patients who underwent endoscopy were rechecked after their initial diagnosis of FE to evaluate the treatment effect. The data regarding general information and clinical characteristics, such as age groups or recheck times, were analyzed after every therapy cycle (Tables 4 and 5). In the regression analysis, age was found to have an effect on the therapeutic outcome (*P* = 0.026). After the modification of this factor, we found that fluconazole had a better curative effect than nystatin (OR = 4.047, 95% CI: 1.282~12.772) or simple observation (OR = 8.91, 95% CI: 2.61~30.49). No significant difference was found between the nystatin and observation groups (*P* = 0.125) (Table 6).

## 7. Discussion

Little information about FE trends in China has been available in recent years. Here, we found that, over an almost 10-year period from 2007 to 2017, the detection rate of FE was 0.82%, and the prevalence significantly increased. The highest prevalence occurred from 2010 to 2013 but decreased in the last 4 years. The FE prevalence in immunocompetent individuals has been reported 0.3-8.7% [4, 8, 16], which is consistent with our findings. The number of FE patients continued to increase because of the popularity of gastroendoscopy and the increasing number of people who underwent this type of exam. This reminds us of the importance of maintaining a focus on FE.

In this study, we found that elderly individuals accounted for a high proportion of FE patients. Increasing age is a risk factor for the onset of the disease, as well as an unfavorable factor for treatment. This may be caused by factors such as declining epithelial cell immunity in elderly people. Current research has confirmed that with increasing age, the number of T cells and B cells in the human body decreases, the antigen presentation ability of dendritic cells decreases, and both innate immunity and acquired immune function decrease [12, 17, 18]. Therefore, elderly people are susceptible to infectious diseases such as FE. In addition, older people often have multiple comorbidities and take combined medications, making the situation more complicated.

In our study, the proportion of patients who had dysphagia or foreign body sensation, which have been previously reported as typical presenting symptoms of FE, was significantly higher in the FE patient group than in the control group. Epigastric pain was more common in the control group than in the FE patient group. This is not surprising since epigastric pain is the most common complaint leading to endoscopy. For comorbidities, we found that malignant tumors were more frequent in FE patients than in control patients, while HBV, cirrhosis, high blood pressure, and diabetes were found equally in both groups. Thus far, there is concern that malignant tumors or chemotherapy will lead to immune suppression, resulting in Candida infection [19]. Additionally, since malignancy and infection share some common risk factors, such as comorbidities, lifestyle factors, and immune suppression, infection can be a risk factor for tumors [20].

Although many different studies have explored the risk factors of FE, only the effect of HIV infection, especially the decrease in CD4 + T lymphocytes < 200/*μ*l, has been consistently demonstrated [21, 22] [23]. Here, we found that the use of antibiotics or immunosuppressants independently contributed to esophageal candidiasis, which is in line with its nature as an opportunistic infection. Some prior studies have found that acid inhibitors, especially PPIs, are a risk factor for esophageal infection [24–27] due to fungal development in the stomach as well as the increase in gastric regurgitation [28, 29]. In contrast, in our study, previous use of PPIs was not associated with FE. Consistent with our data, in a case-control study by Choi et al. and Takahashi et al., PPI use was not associated with FE [9, 11]. However, the control group in our study was composed of patients with some gastrointestinal symptoms, such as epigastric pain. Therefore, most of them received PPI therapy; thus, unmeasured confounders might exist.

Notably, cigarette smoking was also a risk factor for FE in the multivariate analysis. To date, smoking has been demonstrated to disrupt the proliferation and transformation of immune cells and downregulate the secretion of cytokines, thereby inhibiting innate and adaptive immunity. This enhances the pathological inflammatory response and inhibits normal anti-infection immunity [30]. Abdelhabib et al. found that cigarette smoking directly increased the ability of membrane attachment and induced the formation of biological membranes in *Candida albicans* [31, 32].

The comparison of different therapeutic strategies showed that fluconazole had a particularly positive effect on improving the esophageal appearance, while nystatin was also effective. Fluconazole at a dosage of 200 to 400 mg/d for 7 to 14 days is recommended for fungal infections by the guidelines in China, the United States, and Taiwan [22, 33–35]. Nystatin is reported to be effective at a dosage of 3 million U/d for 2 weeks [36, 37]. Nystatin acts by direct contact with the fungus and needs to be swallowed slowly after being dissolved in water; this process may reduce patient compliance. In the fungal culture and drug susceptibility tests of 36 patients, we found that *Candida albicans* was the causative agent, and all the specimens were sensitive to antifungal agents.

The study has the following limitations. In the retrospective case-control study, the information we collected was inevitably influenced by patients' unclear memory, tending to increase the risk of recall bias. In regard to the treatment effect evaluation, only patients with completed therapeutic information were included, resulting in the data loss of others who received therapy elsewhere or without reevaluation, likely increasing the risk of selection bias. As a study in a single-center, these results might elaborate only a single aspect of FE, and a well-designed multicenter study would be helpful to better understand this issue.

In conclusion, this study found that *Candida albicans* was the causative pathogen in patients with FE in South China, without apparent drug resistance. “Dysphagia” and “foreign body sensation” were the most common symptoms and had a certain suggestive effect for this disease. Increased age, smoking, and the use of antibiotics, corticosteroids, or immunosuppressive agents were independent risk factors for FE. Although fluconazole has a good therapeutic effect on fungal esophagitis, we still should pay more attention to FE treatment, given that individuals in today's rapidly aging society and elderly individuals tend to have comorbidities and take multiple drugs.

## Figures and Tables

**Table 1:** Characteristics of FE patients and controls.

Characteristics	*n* = 303
Sex, n (%)	
Male	183 (60.4)
Female	120 (39.6)
Age, n (%)	
≤30	57 (18.8)
31~40	65 (21.4)
41~50	72 (23.8)
51~60	53 (17.5)
>60	56 (18.5)
Comorbidities, n (%)	
Esophageal varices	40 (13.2)
Esophageal polyps	10 (3.3)
Bile reflux	11 (3.6)
Reflux esophagitis	20 (6.6)
HBV	48 (15.8)
Cirrhosis	43 (14.2)
Diabetes	23 (7.6)
HBP	32 (10.6)
Malignant tumor	26 (8.6)
Symptoms, n (%)	
Epigastric pain	88 (29.0)
Chest tightness	19 (6.3)
Acid regurgitation	32 (10.6)
Belching	30 (9.9)
Heartburn	13 (4.3)
AD	58 (19.1)
Nausea	22 (7.3)
Vomiting	15 (5.0)
Hiccups	11 (3.6)
Globus sensation	33 (10.9)
Previous medication, n (%)	
Antibiotics	53 (17.5)
PPI	94 (31.0)
IS	11 (3.6)
Smoking	79 (26.1)

HBV: hepatitis B virus; HBP: high blood pressure; AD: abdominal distention; PPI: proton-pump inhibitor; IS: immunosuppressant.

**Table 2:** Comparison of the clinical characteristics between FE patients and controls.

Clinical characteristics	Controls (*N* = 202)		FE patients (*N* = 101)		*P* value
	*n*	%	*n*	%	
Sex, male	112	55.4	71	70.3	0.01
Comorbidities					
Esophageal varices	28	13.9	12	11.9	0.63
EP	3	1.5	7	6.9	0.03
Bile reflux	10	5.0	1	1.0	0.16
Reflux esophagitis	14	6.9	6	5.9	0.74
HBV	31	15.3	17	16.8	0.74
Cirrhosis	29	14.4	14	13.9	0.91
Diabetes	12	5.9	11	10.9	0.13
HBP	18	8.9	14	13.9	0.19
Malignant tumor	10	5.0	16	15.5	<0.01
Symptoms					
Epigastric pain	68	33.7	20	19.8	0.01
Chest tightness	13	6.4	6	5.9	0.87
Acid regurgitation	23	11.4	9	8.9	0.51
Belching	22	10.9	8	7.9	0.41
Heart burn	9	4.5	4	4.0	1.00
AD	35	17.3	23	22.8	0.26
Nausea	13	6.4	9	8.9	0.43
Vomiting	8	4.0	7	6.9	0.26
Hiccup	8	4.0	3	3.0	0.91
Globus sensation	11	5.4	22	21.8	<0.01
Previous medication					
Antibiotics	27	13.4	26	25.7	0.01
PPI	65	32.2	29	28.7	0.54
IS	2	1.0	9	8.9	<0.01
Smoking	40	19.8	39	38.6	<0.01

EP: esophageal polyps; HBV: hepatitis B virus; HBP: high blood pressure; AD: abdominal distention; PPI: proton-pump inhibitor; IS: immunosuppressants.

**Table 3:** Univariate and multivariate regression analyses of the factors related to FE.

Variables	Univariate analysis		Multivariate analysis	
	Odds ratio (95% CI)	*P* value	Odds ratio (95% CI)	*P* value
Sex, male	1.755 (1.054, 2.921)	0.031		
Age group				0.018
≤30	0.184 (0.078, 0.436)	0.000	0.203 (0.077, 0.535)	0.001
31~40	0.385 (0.183, 0.810)	0.012	0.410 (0.177, 0.953)	0.038
41~50	0.407 (0.198, 0.837)	0.015	0.388 (0.173, 0.870)	0.022
51~60	0.446 (0.206, 0.966)	0.041	0.354 (0.143, 0.873)	0.024
>60	1 (ref)			
Symptom of globus sensation	4.835 (2.239, 10.441)	0.000	7.507 (3.156, 17.860)	0.000
Symptom of epigastric pain	2.055 (1.163, 3.633)	0.013		
Comorbidity of EP	4.940 (1.249, 19.529)	0.023	8.732 (2.006, 38.013)	0.004
Comorbidity of diabetes	1.935 (0.822, 4.554)	0.130		
Comorbidity of malignancy	3.614 (1.575, 8.291)	0.002	4.031 (1.562, 10.407)	0.004
History of IS use	9.783 (2.072, 46.180)	0.004	6.525 (1.089, 39.105)	0.040
History of antibiotic use	2.247 (1.230, 4.105)	0.008	2.178 (1.078, 4.400)	0.030
Smoking	2.548 (1.500, 4.325)	0.001	3.017 (1.645,5.533)	0.000

EP: esophageal polyps; IS: immunosuppressant.

**Table 4:** Characteristics of the therapeutic cases.

Characteristics	*n* = 157
Sex, n (%)	
Male	114 (72.6)
Female	43 (27.4)
Age, n (%)	
≤30	15 (9.6)
31~40	23 (14.6)
41~50	36 (22.9)
51~60	47 (29.9)
>60	36 (22.9)
Initial severity, n (%)	
Kodsi 1	36 (22.9)
Kodsi 2	67 (42.7)
Kodsi 3	46 (29.3)
Kodsi 4	8 (5.1)
Location	
Upper	24 (15.3)
Middle	15 (9.6)
Lower	12 (7.6)
Upper and middle	30 (19.1)
Lower and middle	27 (17.2)
Upper and lower	3 (1.9)
Whole	46 (29.3)
Therapy strategy	
Observation	36 (22.9)
Nystatin	54 (34.4)
Fluconazole	57 (36.3)
Combination	10 (6.4)
Recheck time	
<3 months	69 (43.9)
3~6 months	24 (15.3)
6~12 months	17 (10.8)
>12 months	47 (29.9)
Therapeutic effect	
Effective	125 (79.6)
Ineffective	32 (20.4)

**Table 5:** Comparison of the clinical characteristics between the effective and ineffective groups.

Clinical characteristic	Effective (*N* = 125)		Ineffective (*N* = 32)		*P* value
	*n*	%	*n*	%	
Sex					0.92
Male	91	72.8	23	71.8	
Female	34	27.2	9	28.2	
Age group					0.07
≤30	13	10.4	2	6.2	
31~40	20	16.0	3	9.4	
41~50	33	26.4	3	9.4	
51~60	32	25.6	15	46.9	
>60	27	21.6	9	28.1	
Initial severity					0.26
Kodsi 1	25	20.0	11	34.4	
Kodsi 2	53	42.4	14	43.7	
Kodsi 3	40	32.0	6	18.8	
Kodsi 4	7	5.6	1	3.1	
Location					0.20
Upper	19	15.2	5	15.6	
Middle	12	9.6	3	9.4	
Lower	10	8.0	2	6.2	
Upper and middle	27	21.6	3	9.4	
Lower and middle	24	19.2	3	9.4	
Upper and lower	3	2.4	0	0	
Whole	30	24.0	16	50	
Therapy strategy					0.01
Observation	23	18.4	13	40.6	
Nystatin	41	32.8	13	40.6	
Fluconazole	52	41.6	5	15.6	
Combination	9	7.2	1	3.2	
Recheck time					0.06
<3 months	54	43.2	15	46.9	
3~6 months	19	15.2	5	15.6	
6~12 months	10	8.0	7	21.9	
>12 months	42	33.6	5	15.6	

**Table 6:** Univariate and multivariate regression analyses of the factors related to therapeutic effectiveness.

Variables	Univariate analysis		Multivariate analysis	
	Odds ratio (95% CI)	*P* value	Odds ratio (95% CI)	*P* value
Age stratification		0.091		0.026
≤30	2.167 (0.408, 11.497)	0.364	3.292 (0.557, 19.461)	0.189
31~40	2.222 (0.532, 9.275)	0.273	3.425 (0.748, 15.675)	0.113
41~50	3.667 (0.902, 14.901)	0.069	5.405 (1.235, 22.663)	0.025
51~60	0.711 (0.269, 1.880)	0.492	0.757 (0.265, 2.164)	0.604
>60	1 (ref)		1 (ref)	
Initial severity		0.263		
Kodsi 1	1 (ref)			
Kodsi 2	1.666 (0.663, 4.187)	0.278		
Kodsi 3	2.933 (0.964, 8.929)	0.058		
Kodsi 4	3.080 (0.337, 28.134)	0.319		
Therapy strategy		0.017		0.004
Observation	1 (ref)		1 (ref)	
Nystatin	1.783 (0.708, 4.486)	0.220	2.202 (0.802, 6.043)	0.125
Fluconazole	5.878 (1.876, 18.421)	0.002	8.912 (2.605, 30.490)	0.000
Combination	5.087 (0.578, 44.778)	0.143	7.248 (0.756, 69.435)	0.086
Recheck time		0.081		
<3 months	1 (ref)			
3~6 months	1.056 (0.338, 3.298)	0.926		
6~12 months	0.397 (0.129, 1.219)	0.107		
>12 months	2.333 (0.785, 6.936)	0.127		

## Data Availability

The datasets generated during the current study are available from the corresponding author on reasonable request.
